# Prevalence and Detection of Heteroresistant Vancomycin-Intermediate Staphylococcus aureus (hVISA) Among Clinical Isolates: A Comparative Evaluation of Screening Methods

**DOI:** 10.7759/cureus.88122

**Published:** 2025-07-16

**Authors:** Aditi Agarwal, Lipika Singhal, Ivneet Kour, Parakriti Gupta, Navreet Kaur, Dipanshu Vasesi, Varsha Gupta

**Affiliations:** 1 Microbiology, Geetanjali Medical College and Hospital, Udaipur, IND; 2 Microbiology, Government Medical College & Hospital, Sector 32, Chandigarh, IND; 3 Microbiology, Maharishi Markandeshwar Medical College and Hospital, Solan, IND

**Keywords:** antimicrobial resistance, heteroresistant staphylococcus aureus (hvisa), mrsa (methicillin-resistant staphylococcus aureus), staphylococcus aureus, vancomycin-intermediate staphylococcus aureus (visa)

## Abstract

Background: *Staphylococcus aureus* is a major human pathogen, with methicillin-resistant *S. aureus* (MRSA) posing a significant challenge due to its resistance to multiple antibiotics. Vancomycin remains the drug of choice for MRSA infections; however, resistance mechanisms such as vancomycin-intermediate *S. aureus* (VISA) and heteroresistant VISA (hVISA) have emerged, leading to treatment failures. This study aimed to determine the prevalence of hVISA among clinical isolates and compare the efficacy of different screening methods.

Methods: A prospective, observational study was conducted over 1.5 years, analyzing 102 non-duplicate clinical isolates of *S. aureus* from various specimens. Identification and antimicrobial susceptibility testing were performed using standard microbiological methods. MRSA detection was done using cefoxitin disc diffusion, while vancomycin susceptibility was assessed using minimum inhibitory concentration (MIC) determination. hVISA screening was performed using four methods: brain heart infusion agar with 4 µg/mL vancomycin (BHIV4), gradient plate method (GPM), macro E-test (MET), and E-test GRD, as these methods have been shown to be more effective than other screening techniques in previous studies. The PAP-AUC ratio was used as the gold standard for hVISA confirmation.

Results: Among the 102 isolates, 49.02% were MRSA, and all were susceptible to vancomycin based on MIC values. However, 8.82% exhibited the hVISA phenotype out of the total *S. aureus* (102) isolates. hVISA was more prevalent in hospital-acquired infections (100% of hVISA isolates) than in community-acquired cases. The BHIV4 method showed the highest sensitivity (88.89%), while the gradient plate method had the highest specificity (94.62%).

Conclusion: The detection of hVISA is critical for optimizing treatment strategies and preventing therapeutic failures. The study suggests that BHIV4 screening can serve as an effective method for initial hVISA detection. Continuous surveillance and judicious antimicrobial use are essential to curb the emergence of resistance.

## Introduction

*Staphylococcus aureus* is one of the dreadful and incessant causes of human infections globally. The infection, when coalesced with antimicrobial resistance, poses a deleterious problem, and methicillin-resistant *S. aureus* (MRSA) has been identified as a serious threat by the Centers for Disease Control and Prevention (CDC). Antimicrobial resistance among *S. aureus* emerged in the mid-1940s with the exhibition of resistance to penicillin, owing to the presence of inducible and extracellular serine β-lactamase BlaZ, whose structural gene (*blaZ*) is carried by the transposon Tn552 or Tn552-like elements [1]. This β-lactamase breaks down the β-lactam antibiotic, leading to its ineffectiveness. The enzyme expression is inducible and is controlled by the BlaI repressor and BlaR sensor. However, the prevailing scenario worsened with the emergence of MRSA merely two years after the introduction of methicillin. This has been attributed to the presence of the Staphylococcal Chromosome Cassette mecI (SCCmecI), encoded by the *mecA* gene, resulting in altered Penicillin-Binding Protein 2 (PBP2) with decreased affinity for semisynthetic penicillins and cephems, thereby exhibiting broad-spectrum resistance. The resistance did not remain restricted to SCCmecI; strains harboring SCCmec allotypes, namely, SCCmecII and SCCmecIII, emerged and paved the way for the ongoing global pandemic in healthcare facilities. This hospital-acquired MRSA (HA-MRSA) was soon followed by community-associated MRSA (CA-MRSA), which contains a more mobile, smaller SCCmec allotype (SCCmecIV) along with Panton-Valentine leukocidin (PVL) toxin and is susceptible to most antibiotics other than β-lactams [2].

This ongoing pandemic of MRSA is one of the major hurdles encountered in clinical practice. MRSA isolates are not merely resistant to penicillin/methicillin; they also exhibit resistance to antibiotics like macrolides, tetracycline, aminoglycosides, and fluoroquinolones. Clinicians are left with treatment options such as vancomycin, tigecycline, linezolid, and ceftaroline. Of all these antimicrobials, vancomycin is the drug of choice for MRSA infections. However, with increased and indiscriminate usage, resistance to vancomycin has also emerged, and the first case was reported in 2002 from Michigan, USA. Though cases of vancomycin-resistant *Staphylococcus aureus* (VRSA) have been reported, vancomycin-intermediate *Staphylococcus aureus *(VISA) remains a relatively common entity. VRSA is a consequence of the presence of operons like vanA, resulting in the production of low-affinity precursors D-Ala-D-Lactate or D-Ala-D-Ser, whereas VISA strains exhibit alteration of the cell wall, reducing the diffusion and increasing entrapment of the drug within the cell wall [3].

Besides *S. aureus* strains that are fully resistant, some strains exhibit phenotypic susceptibility to vancomycin (with susceptible MICs) but contain subpopulations with higher MICs, requiring more extensive testing for detection. These strains are referred to as “heterogeneous VISA” (hVISA) and pose an eight-fold or greater inhibitory effect with an uncertain clinical response. Few studies have been conducted in our region to determine the prevalence of hVISA strains, using the gold standard PAP-AUC ratio method compared to screening tests. We also determined the susceptibility pattern of these isolates to older and newer antibiotics. One such antibiotic class includes macrolides, lincosamides, and streptogramins, all of which act on the 50S subunit of the ribosome. Since they have been extensively used, resistance is widespread, and resistance to one class is often linked to the others due to their shared site of action. The genes most commonly involved are the erm gene, which causes target site modification and results in macrolide-lincosamide-streptogramin B (MLS_B_) resistance, and the *msr *gene, which is responsible for the efflux mechanism, causing only macrolide resistance [4].

## Materials and methods

This was a prospective, observational study conducted over one and a half years. A total of 102 non-duplicate routine clinical isolates received from wards, ICUs, and OPDs for routine bacteriological culture were included in the study only after approval from the Institutional Ethical Committee (IEC). Demographic, clinical, and outcome details of patients infected with *S. aureus* were noted. All the strains isolated within 48 hours of admission to the healthcare facility, with no prior history of hospitalization or admission to a nursing home or skilled nursing facility, no previous history of infection with *S. aureus*, indwelling catheters, dialysis, or surgery in the last year of the infection, were considered community-acquired, while the rest were considered hospital-acquired. The identification and susceptibility testing were performed using standard methods. Antimicrobial susceptibility was done using the Kirby-Bauer method of disc diffusion for penicillin, cefoxitin, gentamicin, erythromycin, tetracycline, doxycycline, minocycline, ciprofloxacin, gatifloxacin, norfloxacin, ofloxacin, sparfloxacin, clindamycin, chloramphenicol, and linezolid, while MIC determination using E-strip was performed for vancomycin (HiMedia, 0.016-256 µg/mL), teicoplanin (Liofilchem Diagnostics, VA/TEC 0.5-32/0.5-32 mg/L, 0.016-256 µg/mL), dalbavancin (Liofilchem Diagnostics, 0.002-32 mg/L), telavancin (Liofilchem Diagnostics, 0.016-256 mg/L), tedizolid (Liofilchem Diagnostics, 0.002-32 mg/L), and daptomycin (HiMedia, 0.016-256 µg/mL). MRSA detection was done using a cefoxitin disc (surrogate marker), vancomycin resistance was determined using vancomycin screen agar (VSA), and inducible clindamycin resistance (ICR) was determined by D-zone detection. Zone diameters and MIC values were interpreted according to the Clinical Laboratory Standards Institute (CLSI) guidelines [5].

hVISA screening was done using four methods, namely brain heart infusion agar plates containing 4 μg/mL vancomycin (BHIV4), the gradient plates method (GPM), the macro E-test (MET), and the E-test GRD (glycopeptide resistance detection). hVISA was confirmed using the gold standard population analysis profile-area under curve (PAP-AUC) ratio, for which area under the curve (AUC) was calculated using GraphPad Prism (version 8.0.1) software.

In the BHIV4 method [6], 10 µL of 0.5 McFarland bacterial broth suspensions were inoculated onto brain heart infusion agar plates containing 4 μg/ml vancomycin and incubated at 35 °C along with positive controls (Mu3 for hVISA, Mu50 for VISA) and a negative control (*S. aureus* ATCC 29213). Absence of growth indicated susceptibility to vancomycin; any growth indicated reduced vancomycin susceptibility. If growth appeared at 24 hours, the isolate was considered VISA, and if growth appeared at 48 hours, it was considered hVISA.

**Figure 1:**
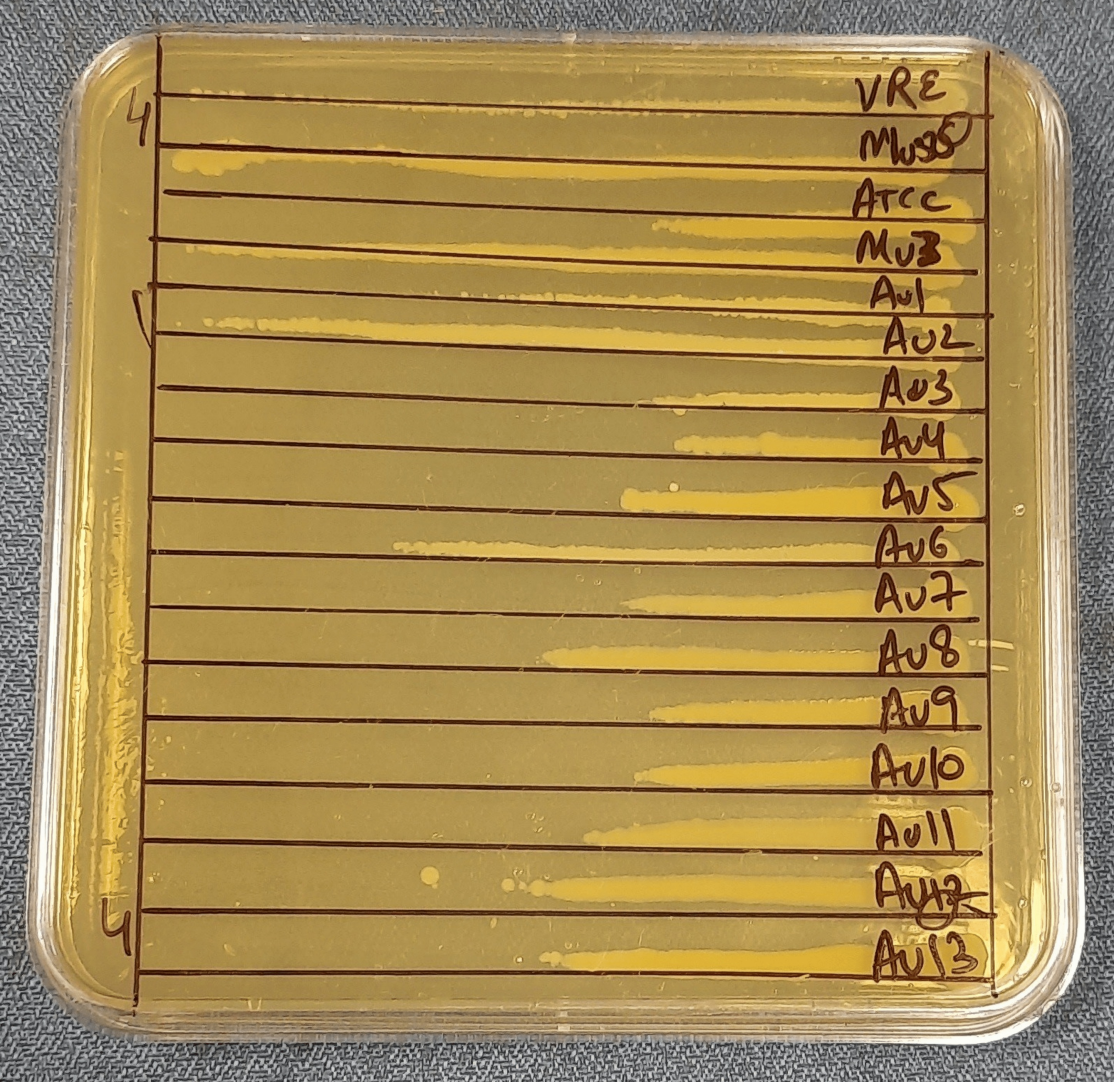
Gradient plate method: screening test for heteroresistant vancomycin-intermediate Staphylococcus aureus (hVISA)

In the gradient plates method (GPM) [6], gradient plates containing a continuous vancomycin concentration from 4.4 μg/mL to 0 μg/mL were prepared in 10 cm square Petri dishes. A standard loopful (10 µL) of a 0.5 McFarland-adjusted inoculum from 10 isolates, along with controls (Mu3 for hVISA and Mu50 for VISA), was streaked across each gradient plate and incubated at 35 °C for 48 hours. Bacterial growth was measured in centimeters, and the ratio of the growth length of the test isolate to the Mu3 control was calculated. A ratio of greater than or equal to 1 denoted an hVISA phenotype, as shown in Figure 1.

In the macro E-test (MET) [6], a bacterial suspension adjusted to 2 McFarland standard was prepared and inoculated onto BHIA and allowed to dry. Vancomycin and teicoplanin E strips were applied, and incubation at 35°C was carried out for 48 hours, after which MICs were observed and interpreted as positive for hVISA if any visible growth was seen at ≥8 μg for both vancomycin and teicoplanin or ≥12 μg for teicoplanin alone.

In the E-test GRD [6], a bacterial suspension adjusted to 0.5 McFarland standard was prepared and inoculated onto blood agar and allowed to dry. GRD E strips were applied, and the plates were incubated at 35°C. The test was interpreted as positive for hVISA if any visible growth was seen at ≥8 μg for vancomycin or teicoplanin.

## Results

A total of 102 non-duplicate isolates of *S. aureus* were included in the study, of which the majority were isolated from pus samples (70.59%), followed by body fluids (13.73%), blood (5.88%), urine (2.94%), CSF (1.96%), sputum and tracheal aspirate (0.98% and 0.98%, respectively), and a few other samples like corneal scraping and skin swabs (2.94%). Of all the cases, 68.63% of patients had HA infection, while CA infection was noted in 31.37%. The mean age was noted to be 43.06 years (median: 43 years), which was comparable in MRSA, MSSA, CA, and HA groups, being 37, 43, 33.5, and 44 years, respectively. The male-to-female ratio was 1.27, and a slight male predominance (55.78% as compared to 44.12%) was reported. The majority (58.82%) were isolated from wards, followed by the Outpatient Department (OPD) (31.37%) and the Intensive Care Unit (ICU) (9.80%), and this distribution was similar for both MRSA and MSSA isolates.

Maximum susceptibility was seen for tedizolid, vancomycin, teicoplanin, dalbavancin, telavancin, and daptomycin, to which all the isolates (100%) were susceptible. These were followed by linezolid (99.02%), chloramphenicol (96.08%), tetracyclines (99.02-84.31%), gentamicin (62.75%), clindamycin (61.76%), cefoxitin (50.98%), cotrimoxazole (45.10%), erythromycin (42.20%), and fluoroquinolones (6.8-17.6%). The isolates were least susceptible to penicillin, for which all the isolates showed resistance (0% susceptibility). Nearly half (49.02%) of the *S. aureus* isolates were found to be methicillin-resistant. Astoundingly, one isolate was found to be resistant to linezolid by the disc diffusion method, although it was susceptible to tedizolid. Among the MLS_B_-resistant isolates, 29.73% showed constitutive and 70.27% showed inducible resistance (as tested by ICR).

We compared the susceptibility profile of MRSA and MSSA isolates, and comparatively higher resistance to all antimicrobials, except cotrimoxazole and chloramphenicol, was noted among MRSA isolates. However, decreased susceptibility to cotrimoxazole (including intermediate and resistant strains) was more prevalent in MRSA strains, despite resistance being higher in MSSA strains.

All the isolates were reported as susceptible to vancomycin, and none of the strains were VISA or VRSA; however, the hVISA phenotype was found in 8.82% of isolates. The distribution of hVISA was comparable among MSSA and MRSA isolates, with hVISA present in 4% of MRSA (1.96% of total samples) and 13.46% of MSSA (6.86% of total samples) isolates. hVISA was reported to be significantly higher in HA isolates. The difference between MSSA and MRSA was not significant (p value <0.5), and all nine hVISA isolates belonged to the HA subgroup.

Among the four screening methods, BHIV4 had the highest sensitivity of 88.89%, followed by the Gradient Plate Method (66.67%), Macro E-test (66.67%), and GRD E-test (44.44%). In the prediction of hVISA, the GRD E-test had the lowest sensitivity of 44.44%. On the other hand, the Gradient Plate Method had the highest specificity of 94.62%, followed by the Macro E-test (89.25%), GRD E-test (88.17%), and BHIV4 (87.10%). In the prediction of hVISA, BHIV4 had the lowest specificity of 87.10%. The highest positive predictive value was found in the Gradient Plate Method (54.55%), and the highest negative predictive value was found in BHIV4 (98.78%). The distribution of hVISA was comparable between MRSA and MSSA and was found not significant (4% vs 13.46%, respectively) (p value = 0.161). There is always a trade-off between sensitivity and specificity (any increase in sensitivity will be accompanied by a decrease in specificity), so we selected the variable in which the combination of sensitivity and specificity provided the maximum predictive value. Overall, BHIV4 was the best predictor of hVISA. The presentation of the categorical variables was done in the form of numbers and percentages. On the other hand, the quantitative data were presented as the means ± SD and as the median with 25th and 75th percentiles (interquartile range). The data normality was checked using the Kolmogorov-Smirnov test. In cases where the data were not normal, non-parametric tests were used. The comparison of the variables that were quantitative and not normally distributed was analyzed using the Mann-Whitney test (for two groups). The comparison of the variables that were qualitative in nature was analyzed using the Chi-square test, and if any cell had an expected value of less than 5, Fisher’s exact test was used. Sensitivity, specificity, positive predictive value, and negative predictive value of BHIV4, GPM, GRD E-test, and MET were calculated for predicting hVISA after taking the PAP-AUC ratio as the gold standard. The data entry was done in the Microsoft EXCEL spreadsheet, and the final analysis was performed using IBM SPSS Statistics for Windows, Version 21 (Released 2012; IBM Corp., Armonk, New York).

## Discussion

*S. aureus *is one of the deadliest microbes in terms of antimicrobial resistance and has been included in the updated list of priority pathogens by WHO 2024 as a high-priority organism (MRSA) [7]. Besides methicillin resistance, vancomycin resistance is also an emerging cause of concern and requires stringent surveillance and detection.

In our study, the majority were pus samples collected from various sites (70.59%), followed by body fluids (13.73%), blood (5.88%), urine (2.94%), CSF (1.96%), sputum and tracheal aspirate (0.98% each), and a few other samples (2.94%). Similar patterns were observed in MSSA and MRSA as well as in CA and HA samples. When the distribution of hVISA was compared between MRSA and MSSA, it was found to be not significant (4% vs 13.46%, respectively; p value = 0.161). Another study conducted earlier at our institute reported similar findings, with skin and soft tissue infections (SSTI) being the most common infection (86.6%) [8]. This corresponds to the fact that SSTI is the most common infection caused by *S. aureus*, which may range from superficial infections like folliculitis, furuncles, and impetigo to deep entities like abscesses and pyomyositis [9]. The findings are in concordance with various studies in which pus was found to be the most common sample for both MRSA and MSSA [10-12].

**Figure 2:**
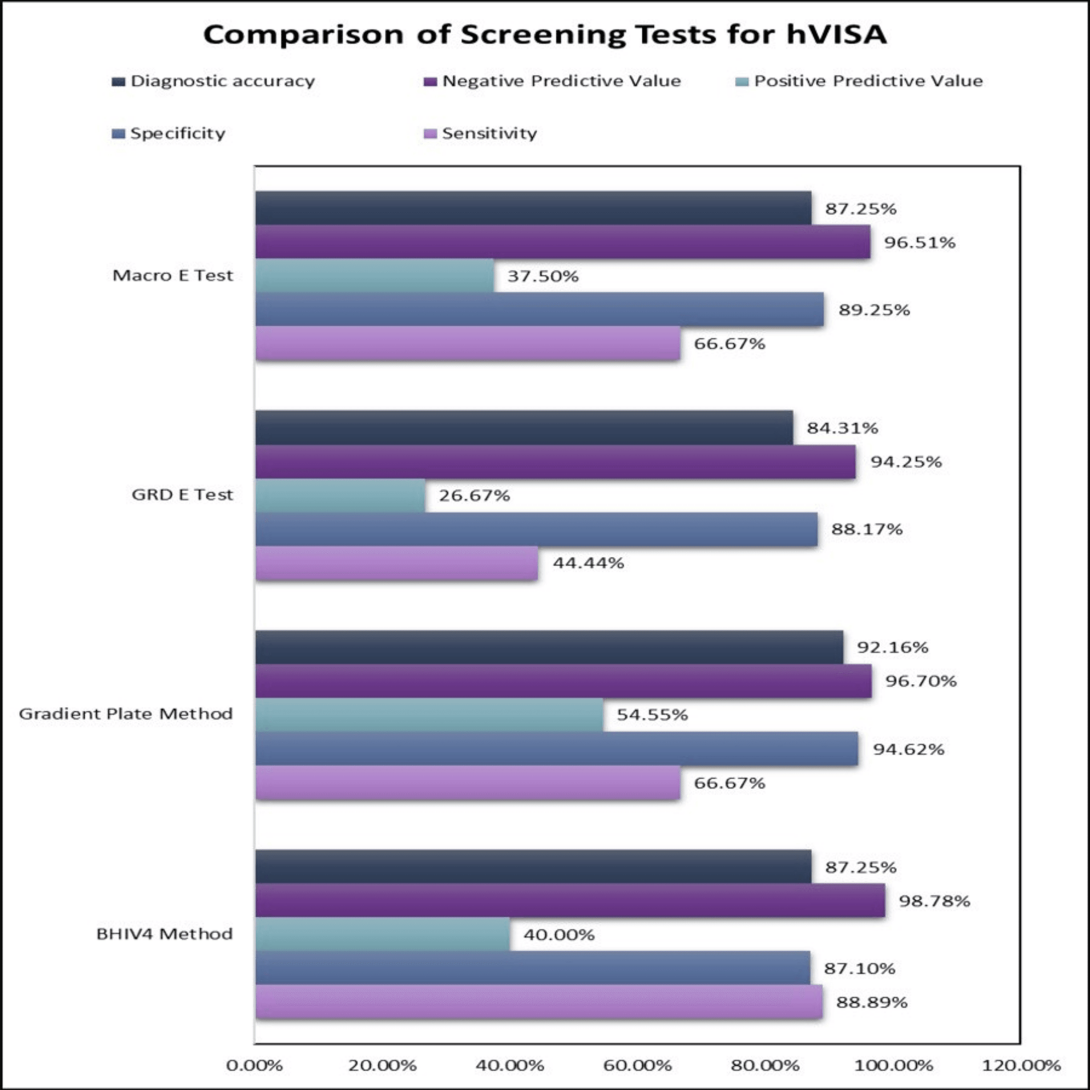
Sensitivity, specificity, positive predictive value, negative predictive value of BHIV4 Method, Gradient Plate Method, GRD E Test, Macro E Test for predicting hVISA after taking PAP-AUC ratio as gold standard. BHIV4: brain heart infusion agar with 4 µg/mL vancomycin, GRD E test: glycopeptide resistance detection E test, Macro E test: macro method E test, hVISA: heterogeneous vancomycin-intermediate *Staphylococcus aureus*, PAP-AUC: population analysis profile–area under the curve.

In the current study, 68.63% of patients had HA infection, while CA infection was seen in 31.37% (Figure 2). Similar numbers were reported in a study by Alvarez-Uria et al. in Andhra Pradesh, where 64.7% of CA infections were reported, while the rest were HA [13]. The high number of HA infections could be due to collection bias, as during the COVID pandemic and under government regulations, most samples were collected during lockdown periods when OPD services were restricted.

In our study, maximum resistance was observed for penicillin (100%), followed by fluoroquinolones (91-64%), cefoxitin (49.02%), erythromycin (38.24%), cotrimoxazole (37.25%), clindamycin (35.29%), gentamicin (26.47%), tetracyclines (11.76-0.98%), chloramphenicol (2.94%), and linezolid (0.98%), in that order. All isolates were susceptible to tedizolid, vancomycin, teicoplanin, dalbavancin, telavancin, and daptomycin. In a study conducted at the same institute by Gupta et al., similar resistance patterns were observed, with resistance to penicillin (91%) being the highest, followed by ciprofloxacin (62.8%), erythromycin (45.9%), cotrimoxazole (34.9%), and clindamycin (26.7%) in decreasing order [8]. In a study published in 2020, Gurung et al. described a slightly different pattern of resistance, where penicillin and cefoxitin resistance were highest, followed by azithromycin, ciprofloxacin, erythromycin, tetracycline, cotrimoxazole, clindamycin, chloramphenicol, gentamicin, and vancomycin in that order [14].

We found that all the strains were resistant to penicillin. According to recent studies, the incidence of resistance to these penicillinase-labile penicillins is quite high, presenting as 100% resistance in some locations and more than 90% in various other studies, which is in concordance with our findings [10,12]. However, another study by Cheng et al. from Canada described 28% of penicillin-susceptible isolates from bloodstream infections caused by MSSA, which was described as a re-emergence of penicillin susceptibility [15].

In our study, nearly half (49.02%) of the *S. aureus* isolates were found to be methicillin-resistant (MRSA). A systematic review conducted during 2015-2019 on the prevalence of MRSA from various parts of India found the overall prevalence to be 37%, with the highest pooled prevalence from Jammu and Kashmir (55%) and the lowest from Maharashtra (34%) [16].

We found only one linezolid-resistant strain, which, however, was susceptible to tedizolid, a drug belonging to the same class of antimicrobials, i.e., oxazolidinones. Similarly, in the LEADER surveillance program (2011-2015), one linezolid non-susceptible strain was found out of 3031 strains tested over a period of five years [17]. Both tedizolid and linezolid inhibit protein synthesis by binding to the 50S subunit of the ribosome. Tedizolid shows increased potency due to structural differences that engage additional ribosomal binding sites. The cfr gene, which results in multidrug resistance, is transferred by a mobile genetic element and is responsible for resistance to the pleuromutilins, streptogramin A, oxazolidinones, phenicols, and lincosamides. In addition, chromosomal mutations in genes encoding the 50S ribosome can also cause resistance to linezolid.

The tedizolid-susceptible strain can be attributed to its four- to eightfold higher potency compared to linezolid, as well as its activity against *cfr*-gene-mediated linezolid resistance [18]. Tedizolid is, however, not active against 23S rRNA (ribosomal protein) mutations, which are the major mechanism of resistance to linezolid [19]. Hence, linezolid-susceptible strains are considered tedizolid-susceptible, but some linezolid-resistant strains may still be tedizolid-susceptible.

While comparing the antimicrobial resistance among CA and HA strains, comparable patterns were observed in our study for all the agents under study. A similar comparable susceptibility pattern was observed in CA and HA by Preeja et al. in MSSA and MRSA [10, 12], while Alvarez-Uria et al. described slightly higher resistance in HA among gentamicin, doxycycline, norfloxacin, and sparfloxacin resistance prevalence. [13]

On the contrary, on comparison between MRSA and MSSA isolates for the antimicrobial susceptibility, a higher resistance percentage was seen in MRSA for all antibiotics tested except cotrimoxazole and chloramphenicol. Similar patterns were seen in studies such as the one done by Heyar et al. [20] Alvarez-Uria et al. stated that globally, all anti-antimicrobial agents have a higher resistance rate in MRSA, except for chloramphenicol, which is in agreement with our study. [13]

Furthermore, gentamicin and fluoroquinolones (ciprofloxacin, gatifloxacin, and norfloxacin) had significantly decreased susceptibility in MRSA in our study, while strains with decreased susceptibility (intermediate + resistant) to cotrimoxazole were higher in MRSA as compared to MSSA, even though resistant strains were higher in number in MSSA. In a study showing similar patterns of higher resistance to aminoglycosides in MRSA, genes targeting aminoglycosides like gentamicin, tet(K), were found in only 2% of MSSA and 45% of MRSA. [21] The resistance to fluoroquinolones could be associated with the widespread use of this class of drug.

All the strains were susceptible to vancomycin, teicoplanin, dalbavancin, telavancin, and daptomycin in our study. Similar findings with no resistance to one or more of these antimicrobial agents have been described in many studies. [10, 12, 22] Even though these antibacterial agents are largely active against this organism, resistance is emerging in proportion to their usage for MRSA infections. Consequently, the judicious use of these antibiotic agents has to be the priority.

In the present study, all isolates had MICs in the susceptible range for vancomycin; hence, all were VSSA (100%), and none of the strains were VISA or VRSA, while the prevalence of the hVISA phenotype was found to be 8.82%. However, decreased susceptibility from various regions has been reported, and according to a study, the global prevalence of VRSA, VISA, and hVISA isolates was 1.5%, 1.7%, and 4.6%, respectively, while the same for Asia was 1.3%, 2.1%, and 4.7%, respectively. In the same study, India had a prevalence of 1.6%, 4.6%, and 2.5% for VRSA, VISA, and hVISA, respectively. [23] Another study showed a significant increase in the vancomycin MIC during a period of five years of the study, attributing it to the vancomycin creep phenomenon. [24] The data varies according to the area of study, as well as being influenced by the usage of the drug in that area, along with the antibiotic surveillance program and its compliance rates.

In our study, the distribution of hVISA was comparable in MSSA and MRSA, with no significant difference between them. However, the distribution of hVISA was significantly higher in HA as compared to CA isolates, as all nine hVISA isolates belonged to the HA subgroup. This is in agreement with different studies where hVISA and their successor VISA isolates have been associated with HA infections that were resistant to a broad spectrum of antimicrobials compared with VSSA and are also associated with vancomycin treatment failure, persistent bacteremia, longer hospital stays, and adverse final clinical outcomes. [25] Since the development of resistance requires exposure to the drug, and this class of antimicrobial agent is not used outside hospital settings, this association with HA isolates is of paramount importance.

**Figure 3:**
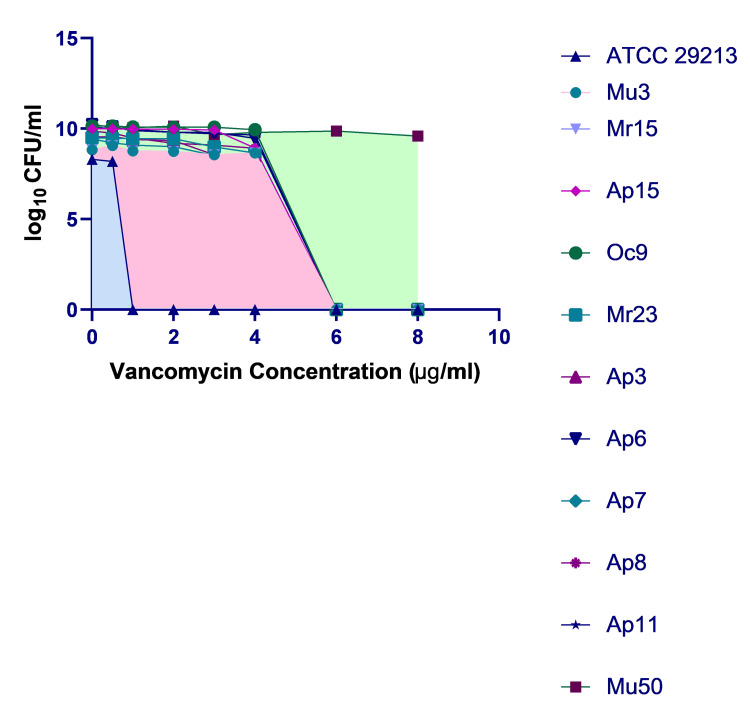
Population analysis profile–area under the curve: confirmatory test for heterogeneous vancomycin-intermediate Staphylococcus aureus (hVISA) Graph showing the concentration of vancomycin (in μg/mL) plotted against the log₁₀ of colony-forming units at that concentration, depicting hVISA isolates along with control strains ATCC 29213, Mu3, and Mu50. The colored area shows the area under the curve of the controls, with ATCC 29213 in blue, Mu3 in pink, and Mu50 in green.

The increasing prevalence of hVISA, coupled with no routine tests for the detection of the same, is worrisome and needs immediate attention. The PAP-AUC method, which is the gold standard for hVISA detection, is cumbersome and cannot be done as a routine procedure in clinical laboratories. [9] So, we need efficient methods to reasonably screen for hVISA, which can quickly predict the possibility of their existence in a sample. Four of these different screening methods were used in this study and compared on various grounds, taking the PAP-AUC ratio as the gold standard (Figure 3). Among these, maximum positivity as well as maximum negative predictive value was seen by the BHIV4 method (19.61% and 98.78%, respectively), making it the best predictor of hVISA among the four methods tested. Similar results were given by Reiderer et al., where the BHIV4 method was reported as more precise compared to E-test-based screening methods. [26] However, Singh et al. suggested MET be used as the best predictor for hVISA on comparing all these methods. [27] The screening agar-based method gives us a feasible, inexpensive, and easy-to-perform option to search for the hVISA strain. A similar method for VRE and VISA/VRSA is already in use and standardized, which could be used as a base to include this method as well in the clinical microbiology laboratories.

## Conclusions

*S. aureus *is one of the most resistant pathogens and is on the list of priority organisms under surveillance programs of national and international interest. Complete resistance to penicillin was seen in our study, while methicillin resistance was seen in nearly half of the isolates. All the isolates were susceptible to tedizolid, vancomycin, teicoplanin, dalbavancin, telavancin, and daptomycin, but 8.82% were found to have a heterogeneous phenotype in the form of hVISA. Since the gold standard method for the detection of hVISA is laborious and cumbersome, a screening method is the need of the hour to implement antimicrobial stewardship as well as to provide appropriate treatment for patients. The development of simple and reliable screening methods, like BHIV4, could be a game-changer for quickly identifying hVISA strains. This is especially important because early detection can help doctors make better treatment decisions, preventing complications and ensuring patients receive the right care sooner. The need of the hour is to conduct studies to validate and standardize such methods, as well as to consider newer ways to tackle resistant strains of *S. aureus*. Lastly, strengthening surveillance programs and antimicrobial stewardship is key to staying one step ahead of these superbugs, ensuring that we do not lose the battle against these infections. Out of the four screening methods tested, BHIV4 was found to be the best predictor of hVISA, providing a simple, easy-to-perform method that can be integrated into existing operating guidelines in our laboratories.
